# Nutritional support for lactating women with or without azithromycin for infants compared to breastfeeding counseling alone in improving the 6-month growth outcomes among infants of peri-urban slums in Karachi, Pakistan—the protocol for a multiarm assessor-blinded randomized controlled trial (Mumta LW trial)

**DOI:** 10.1186/s13063-020-04662-y

**Published:** 2020-09-01

**Authors:** Ameer Muhammad, Yasir Shafiq, Muhammad Imran Nisar, Benazir Baloch, Amna Tanweer Yazdani, Nida Yazdani, Fyezah Jehan

**Affiliations:** 1 VITAL Pakistan Trust, Karachi, Pakistan; 2 Department of Pediatrics and Child Health, Aga Khan University, Karachi, Pakistan

**Keywords:** Balanced energy protein supplements, Lactating women, Azithromycin, Exclusive breastfeeding

## Abstract

**Background:**

Globally, 45% of under-five deaths are either directly or indirectly attributable to malnutrition, and most of these deaths are in low- and middle-income countries (LMICs). Children are particularly vulnerable in the first 6 months of life. An estimated 4.7 million infants under the age of 6 months are moderately wasted, whereas 3.8 million are severely wasted. Although the children of malnourished women have an increased risk of stunting and wasting, there is little information on this issue.

**Methods:**

This is a community-based, open-label, multiarm randomized controlled trial that will include parallel group assignments with a 1:1:1 allocation ratio in low-income squatter settlements in urban Karachi, Pakistan. The women in the control group (control arm) will receive standard counseling only, whereas the lactating women in the first intervention group (intervention arm 1) will receive two sachets of balanced energy protein (BEP) supplementation per day from enrollment until the infant reaches 6 months of age. The lactating women in the second intervention group (intervention arm 2) will receive the same BEP supplementation as those in intervention arm 1 while their babies will also receive a single stat dose (20 mg/kg orally) of azithromycin at 42 days. The primary outcome will be the relative length velocity from 0 to 6 months by intervention arm. The primary analysis will be intention-to-treat analysis.

**Trial registration:**

ClinicalTrials.gov NCT03564652. Registered on 21 June 2018

## Background

Globally, approximately 45% of under-five deaths are attributable to childhood malnutrition, primarily in low- and middle-income countries (LMICs). Moreover, an estimated three million under-five deaths annually are caused by undernutrition [1]. Children are particularly vulnerable in the first 6 months of life, and globally, an estimated 4.7 million infants under the age of 6 months are moderately wasted and 3.8 million are severely wasted [2]. Breastfeeding confers protection, but in the face of maternal undernutrition, infection risk and competition with older siblings for breastfeeding due to the interpregnancy intervals being short can lead to undernutrition [3]. The prevalence of lactating women in LMICs who are underweight themselves is high [4–7], and they require an additional 500 kcal per day as well as iron, iodine, and other micronutrients to maintain milk quality [8–10]. Inadequate maternal dietary intake during pregnancy and lactation is the single strongest predictor of stunting and underweight among children [11]. The implications in terms of child growth, development, and long-term health cannot be overstated [12, 13].

Despite this issue, few randomized controlled trials have been conducted to guide the development of interventions. In the intervention arm of a study conducted in Ghana using small-quantity lipid-based nutrient supplements (SQ-LNS), iron and folic acid were taken (IFA group), and multiple micronutrients (MMN group) were taken daily during healthy pregnancy until delivery and for 6 months postpartum; the infants in the LNS arm had significantly higher length, length-for-age *z*-score (LAZ), weight, and weight-for-age *z*-score (WAZ) values than did the other groups at 18 months of age [14]. These findings, however, were not observed in the study by Lanou et al. in Burkina Faso, who used similar pre- and postpartum interventions [15]. These studies were, however, not sufficiently powered for analyzing the effect in undernourished women, i.e., the mid-upper arm circumference (MUAC) was less than 23.0 cm or the body mass index (BMI) was low. Additionally, most of these trials where perinatal supplementation was given to the mother showed that the impact of these supplements was not sustained during the infant period [16]. Though the MMN study showed only minimal differences in linear growth [15], in theory, macronutrient supplements can support growth.

Additional evidence is therefore required to assess the effectiveness of high balanced energy protein (BEP) supplementation in undernourished lactating women in LMICs. In addition to growth, fetal and infant nutritional compromise directly increases infection risk [17]. Interest in this area has been rekindled by the results of a recent study involving mass azithromycin prophylaxis in young children, showing a reduction in all-cause mortality [18]. Despite theoretical concerns regarding the development of bacterial resistance, there is currently no evidence of the emergence of such strains [18, 19]. Understanding the pathways of how these interventions might work is still not very clear, i.e., if there is any impact on growth, what is the possible explanation [19]? Moreover, we want to assess the impact of these interventions, if any, on inflammatory biomarkers in both stool and blood, iron deficiency, and the composition of breastmilk. The study results will additionally enable us to examine the changes in infant stool microbiota that play a substantial role in inflammation, immune development, enteropathy, and the nutritional-antimicrobial causal pathway [20–23]. Therefore, a gap exists in the current knowledge in the context of standard of care being “only nutrition counseling to the undernourished lactating women”; whether a high-dose BEP supplements (16–21 g of protein per day) during lactation is beneficial for the infant’s growth has yet to be determined. Additionally, whether a combination of BEP with a single prophylactic dose of azithromycin to the infant of the same mother has any additional benefits and has yet to be studied [18, 19]. Furthermore, given that the prevalence of stunting among children under five is around 50% in Pakistan, our primary outcome of interest is length velocity over the period of the first 6 months.

## Objectives

The primary objective is to compare the efficacy of fortified, balanced energy protein (BEP) supplements being consumed by lactating women for 6 months (intervention arm 1) with or without a single prophylactic dose of oral azithromycin to the infant at 42 days of age (intervention arm 2) with that of standard exclusive breastfeeding and nutritional counseling alone (control arm) in improving the length velocity among infants at 6 months (outcome). The secondary outcome is to compare the impact of the interventions on weight (or growth) velocity, *z*-scores, breast milk quality, gut microbiota, and key micronutrient levels in both the mother and infant.

## Methods

### Trial design

This is a multiarm community-based randomized controlled, open-label, assessor-blinded superiority trial with a treatment allocation ratio of 1:1:1. A multiarm trial was selected to determine the incremental impact of BEP to the mother, along with oral azithromycin to the infants, on length velocity.

### Study setting and study population

The trial is being conducted in the peri-urban communities of Karachi, Pakistan. These are impoverished coastal slums with a population of approximately 250,000 residents, according to the census conducted in 2017. The annual birth cohort is approximately 5000 each year. The population is multiethnic and includes Sindhi-, Pashtun-, Punjabi-, Bengali-, and Urdu-speaking individuals. Through a demographic surveillance system (DSS), bi-monthly visits are made to married women of reproductive ages (13–49 years) in the catchment area. During these visits, mortality, pregnancy, in- and outmigration, and the current number of children under 5 are recorded. According to previous studies, there is a high prevalence of low birth weight (30%), stunting (52%), and wasting (18%) in the study areas. The study population includes only lactating women of reproductive ages who have recently delivered their infants.

### Participant eligibility criteria

Lactating women between 13 and 49 years of age and their newborns will be enrolled if they fulfill the inclusion and exclusion criteria provided in Table 1. The mid-upper arm circumference (MUAC) is measured routinely during the surveillance rounds, and the women with a MUAC of less than 23 cm in the first week of delivery are screened for eligibility by a research team. For eligible participants, written informed consent is obtained in the local language.

**Table 1:** Eligibility criteria

Inclusion	Exclusion
Lactating women with a mid-upper arm circumference of < 23.0 cm	Newborns with a birth weight of less than 1500 g
Infants with a live birth outcome, captured within 168 h	Newborns with a known congenital anomaly or other severe illnesses based on the study physician’s assessment before enrollment
Individuals with the intention to stay in the catchment area for the entire duration of trial after enrollment	Lactating women with known allergies to peanuts, lentils, chickpeas, or dairy products
Individuals with the intention to exclusively breastfeed the child for at least 6 months of age	Individuals who were previously enrolled in the trial
Individuals who have provided voluntary written informed consent	

### Sample size

There are limited data available on the impact of BEP on length velocity over the first 6 months of life in infants. However, in one of the studies, the mothers received only perinatal supplement, and the overall increase in length (cm/month) was *b* = 3.289 among the LNS group and *b* = 3.346 among the MMN group [15]. However, the women in this trial received supplements with smaller doses/energy than the women in our study will receive, and the intervention time was also different, i.e., the perinatal period rather than the infancy period. Another study showed that the difference in length velocity (cm/month) was 0.02 between the MMN and IFA groups over a period of 0–18 months [17]. Therefore, in the absence of clear evidence on the impact of these interventions, we hypothesized the effect size to be 0.12 cm/month. This is also based on learning through field experience; when severely malnourished lactating women (MUAC < 19.0 cm) were provided with chickpea-based ready-to-eat supplements (100 g/day for 3 months), the difference was 0.06 cm/month at 3 months of age between these women and women who did not receive any supplements (unpublished data that was used purely for implementation and was not included in a study).

The null hypothesis for this trial is that the mean difference in length velocity between an infant of a lactating woman receiving standard breastfeeding counseling with BEP alone (intervention arm 1) for 6 months or in combination with a single prophylactic dose of azithromycin at 42 days of age (intervention arm 2) and an infant of a lactating woman receiving standard breastfeeding counseling alone (control arm) is equal or less than 0.12 cm/month (primary outcome). Due to the absence of evidence of such interventions on length velocity, we based this hypothesis on our local experience and field data from our field sites. The alternate hypothesis for this trial is that an infant of a lactating woman receiving BEP alone for 6 months or in combination with a single prophylactic dose of azithromycin at 42 days of age has a mean length velocity is greater than 0.12 cm/month that of an infant of a lactating woman not receiving an intervention at 6 months. The sample size takes multiple comparisons into account and is based on the primary outcome of length velocity with an effect size difference of at 0.12 cm per month between the arms, a 1-sided test, power of 80% and an alpha of 0.025 to account for multiple comparisons (the lower alpha). A drop-out rate of 14% (i.e., 10% loss to follow-up and infant mortality rate of 4%) is assumed in the study, so the minimum total sample size required is 957 (319 lactating women in each arm).

### Recruitment

Because married women are included in the surveillance system established by the Department of Pediatrics and Child Health at Aga University, VITAL Pakistan Trust has access to a list of all pregnant women in the catchment area. Using this list, the research team will visit these households, connect with the pregnant women, and leave our contact number so that they can notify us of birth-related events. During each pregnancy touchpoint, the research team will provide standard antenatal and nutrition counseling to each pregnant woman and encourage them to seek proper care. At the time of birth, the randomization/enrollment team will receive a birth notification so that the team can visit the household for an eligibility assessment.

### Informed consent procedure

For the eligible participants, written informed consent will be obtained by the same team in a local language (mostly Urdu and, where required, in the languages Sindhi and Pashto). The team members will explain the details of the trial, including the purpose, follow-up procedures, specimen collections, and other related processes. If a participant is eligible and agrees to undergo the procedure as explained, the research team will give the consent form to the participant or decision-maker, or if they cannot read it, a team member will read it word-by-word for them in Urdu or the local language. The participants will be allowed to ask any questions related to the consent form and trial procedure. If the participant/decision-maker requires additional time to make more informed decisions, the team will also allow this opportunity and wait until the final voluntary decision is made. If the participant voluntarily agrees to participate, the participant will sign the consent form in the presence of a witness; either the form is duly signed or a thumb impression is provided by the participant and the witness. The ethics committee approved the use of a thumb impression by the participant and witness if they cannot read or write. Only the designated study team will be involved in obtaining written informed consent. A copy of informed consent will be provided to all participants and attached in the file with the study ID. Additional analyses are planned, which may involve sending samples and data abroad. The consent document covers all the aspects of these procedures, and the participants have an opportunity to opt out from biobanking or from participating in future research at any point of the trial. Nevertheless, any future secondary analysis will require institutional and national ethics committee approval.

### Randomization and allocation concealment

**Fig. 1:**
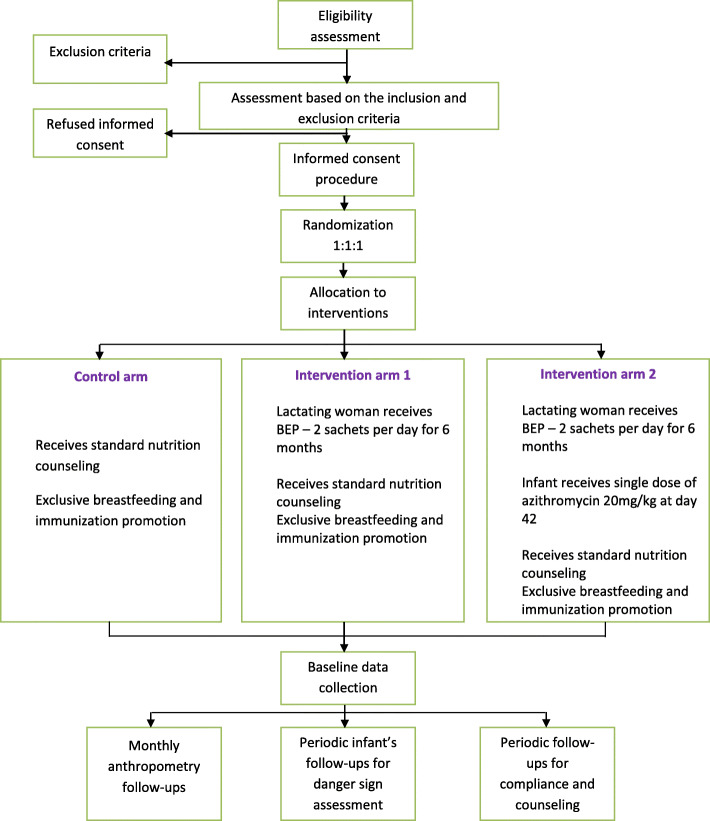
CONSORT flow diagram of the trial

After written informed consent is obtained, randomization will be performed by the team. Stratified block randomization with blocks of sizes 3, 6, and 9 will be used. Sequence generation will be performed by an independent statistician using a random selection method before the beginning of the trial. Self-adhesive, precoded sticky labels with unique identification numbers will be applied to sealed opaque envelopes containing the coded randomization identification number and intervention name to ensure that the randomization process and allocation are blinded. Baseline information regarding nutrition and exclusive breastfeeding will be recorded. Anthropometry measurements of both the mother and newborn will be performed, and follow-up procedures will be explained. Figure 1 shows the trial processes in detail.

### Blinding

The outcome assessors will be blinded, responsible only for the anthropometry measurements, and will be assigned a schedule that does not overlap with those of the follow-up teams. All investigators will also be blinded to group allocation throughout the period of the study. Furthermore, a statistician will independently perform the interim analysis for the Data Safety and Monitoring Board (DSMB) blinded by arm. Furthermore, the data analyst who will perform the final analysis will be blinded, and the code will eventually be revealed after the blinded results are shared with DSMB and investigators in a final review meeting.

### Interventions

In the control arm, the lactating women will receive standard nutritional counseling and promotional messages of exclusive breastfeeding as well as standard of care by a trained research team. In “intervention arm 1,” the lactating women receive, in addition to the above, 2 sachets of BEP supplements per day until the infant reaches 6 months of age, and the sachets will be distributed by a trained research team at enrollment and each follow-up visit. BEP is a certified product of the World Food Program and is locally produced by Ismail Industries in Karachi. The manufacturers do not and will not play a role in any part of the study. Each sachet contains a caloric value of 400 kcal per 75 g and approximately 10.5 g of protein. The sources of protein are mainly chickpea, peanuts, lentils, legumes, and skimmed milk. In “intervention arm 2,” the lactating women receive standard nutritional counseling and promotional messages of exclusive breastfeeding along with BEP supplements (the same as intervention arm 1), but in addition, their infants will also receive a single prophylactic dose of azithromycin oral suspension, 20 mg/kg, at day 42 of life (window period of an additional 7 days). All participants in the arms will receive routine care, including newborn care, immunization, counseling regarding newborn and infant care at home, and timely referral to health facilities in cases of urgent need. All other nonstudy treatments, such as medications and formula milk for infants, will be recorded at each follow-up. In the case of any severe illness or adverse event (reported or observed), the intervention may be paused or stopped for a limited period after the investigators and the Data Safety and Monitoring Board are consulted.

### Data collection and data management

Case report forms (CRFs) are designed to capture details on screening, eligibility, randomization, household demography, newborn assessment, danger signs, severe adverse events, compliance with interventions, exclusive breastfeeding, 24-h food recall (to estimate usual intake as well as diversity on a monthly basis), and anthropometry. The data will be collected on tablets with built-in logic checks and skip patterns by a trained team and updated on secure servers in real-time using digital applications, which are designed and built in-house. Auto alerts will be used to remind the participants about the follow-up schedule, and the data will be tracked according to key indicators. All the data will be collected in a real-time manner and uploaded on a cloud server, which is password protected and only accessible to the trial data management team and manager. Participant confidentiality will be maintained through a unique ID system, and the participant identification information will not be exposed to anyone outside the trial team. The tablets that will be used will be password protected and only accessible to the study team. Deidentified data will be used for analysis purposes.

### Follow-ups

Follow-up teams will perform home visits to provide counseling for all arms. BEP will also be provided (intervention arm 1), and compliance will be measured by logging the number of empty sachets since the last visit. Azithromycin 20 mg/kg stat oral dose (intervention arm 2) will be given to infants at day 42 of life. Compliance with exclusive breastfeeding will be assessed for all arms since the last visit. Follow-ups will be performed daily for the first 15 days on alternate days for 2 weeks, every 72 h for 2 weeks, and, finally, weekly until the child reaches the age of 6 months. Monthly 24-h food recall data will also be collected. At each visit, counseling will be provided to the participants to reinforce adherence to the BEP and exclusive breastfeeding protocols. For participants who move out of the catchment area, the team has developed a system to follow them at the new location, if possible. There is no plan of retention for these participants once their 6-month follow-ups are completed. However, through our existing free-of-cost primary health care facilities, standard of care is available to all participants, even after the completion of the trial. After enrollment (within 168 h of birth), the total follow-up duration of each participant in the trial will be 6 months. Table 2 shows the schedule of enrollment, interventions, and assessments.

**Table 2:** The schedule for enrollment, the interventions, and the assessments

Time points	*T*_0_^*a*^	Follow-ups for data on the key study variables and indicators						Follow-ups for anthropometric measurements	Study endpoint
		Days 1–13^*b*^	Days 14–25	Days 26–38	Days 39–44^*d*^	Days 45–52	Day 53-179	Day 27, 56, 85, 114, and 143	
		Daily visits^*c*^	48 hourly visits	72 hourly visits	Daily visits	72 hourly visits	Weekly	Monthly	Day 179
**Enrollment**									
*Eligibility assessment*	Χ								
*Written informed consent*	Χ								
*Randomization and allocation*	Χ								
*Baseline data*	Χ								
**Intervention**									
*Nutrition and breastfeed counseling*		Χ	Χ	Χ	Χ	Χ	Χ		
*BEP distribution and compliance assessment*^*e*^		Χ	Χ	Χ	Χ	Χ	Χ		
*Oral azithromycin administration at day 42 and daily follow-ups for SAE monitoring after dose administration*^*f*^					Χ				
**Follow-ups**									
*24-h breastfeeding recall*		Χ	Χ	Χ	Χ	Χ	Χ		
*Infant assessment for danger signs*		Χ	Χ	Χ	Χ	Χ	Χ		
*24-h maternal food intake recall*^*g*^							Χ		
*Monthly maternal and infant anthropometry measurements performed by a separate team (blinded from intervention details)*								Χ	Χ
*Specimen collection at two time points only (mother and infants)*^*h*^						X	X		

^a^Window period for eligibility assessment and enrollment is 0–6 days of infant’s life

^b^Age of the infant

^c^Follow-up start day is depending on the age of the infant at the time of enrollment

^d^Pre- and post-azithromycin administration

^e^Only in the intervention arms 1 and 2

^f^Only in intervention arm 2 and window period of 7 days

^g^Only monthly basis

^h^Two time points only—first at day 41 and second at day 56 (window period of 7 days)

### Anthropometry

The teams will be trained to perform anthropometry measurements for the infants and mothers by a master trainer using the INTERGROWTH-21st standards, and they will attend monthly refreshers. The measurements will include the infant length, weight, MUAC, and head circumference, and the maternal MUAC and weight. Two team members will measure the infant and mother, and the team members will be blinded to the other member’s measurements; the data will be entered digitally in the system, which will calculate the average value automatically. For the infant weight, we will use the Laica weighing scale model PS3001, whereas the SECA adult weighing scale model 874 will be used to assess the maternal weight. We imported MUAC tape from UNICEF. The SECA scale, model 417, and SECA scale, model 213, will be used to measure the infant length and maternal height, respectively. The allowable difference between the two measures according to the study standard procedure is ± 0.5 cm for maternal MUAC, ± 0.2 kg for maternal weight, ± 0.5 cm for maternal height, ± 20 g for infant weight, and ± 0.4 cm for infant length, infant MUAC, and infant head circumference. To record growth longitudinally, the same anthropometry measurements will be taken at days 27, 56, 85, 114, 143, and 179 of the infant’s life.

### Primary outcome

The primary outcome of interest will be the length velocity (cm/month) at 6 months. This will be defined as the mean difference in length velocity measured at birth (or baseline) and at 6 months of age, expressed as the change in centimeters per month.

### Secondary outcomes

The other outcomes of interest will be weight velocity (gm/kg/day), length-for-age *z*-score (LAZ), weight-for-age *z*-score (WAZ), and weight-for-length *z*-score (WLZ). Weight velocity (gm/kg/day) will be defined as the mean difference in weight velocity measured at birth (or baseline) and at 6 months of age, expressed as the change in gm/kg/day. Furthermore, the mean differences in the specific *z*-score indicators (LAZ, WAZ, and WLZ) measured at birth (or baseline) and at 6 months of age will be assessed. Furthermore, the anthropometry measurements of the mother will also be assessed, i.e., height (cm), weight (kg), and MUAC (cm), and the mean change in each of these indicators between different arms will be assessed.

### Biomarker assessment (other secondary outcomes)

All laboratory specimens from the mothers and infants will be collected on days 40 and 56 of the infant’s life, i.e., two different time points, which were selected based on the administration of oral azithromycin to the infants in intervention 2 at day 42. Therefore, the first time point (day 40) of specimen collection will be before the azithromycin dose, and the second time point (day 56) will be at 14 days after the azithromycin dose. A window period of an additional 7 days will be allowed for specimen collection, depending on the delay, if any, in the administration of the azithromycin dose to the infant. To standardize the arms, the same time points for specimen collection will be used for all the arms. Furthermore, to complete the infant-mother dyad, the same mother’s specimens will also be collected at the same time points. The differences in the mean values (all continuous variables) of the specimens collected at the second time point (day 56) are of primary interest, and comparisons will be made with the values of specimens collected at the first time point, i.e., the baseline measurement. Further, comparisons will be made within the arm (second time point compared to baseline) between the arms as well as the mother-infant dyad. Furthermore, we will also calculate the interquartile ranges for similar comparisons as mentioned above.

For the laboratory procedures, blood samples will be collected from all infants whose parents/caregivers have provided consent for hemoglobin (gm/dl), ferritin (ng/ml), transferrin (mg/dl), C-reactive protein (CRP) (mg/l), and alpha1-acid glycoprotein (AGP) (mg/ml) tests. The rationale for assessing the levels of hemoglobin, ferritin, and transferrin is to determine whether there are any differences in the markers for iron deficiency anemia in the infants across arms. Hemoglobin assessment will be performed with Hemacue equipment, while ferritin and transferrin will be assessed using an immune-turbidimetric assay with a Roche Cobas c-311 automated clinical chemistry analyzer. AGP and CRP will be analyzed using the same assay and equipment to observe differences in these inflammatory biomarkers among the three arms. To complement and link the findings of the infant biomarkers with the maternal intervention, 50 lactating women from each arm will also be approached at the same time point to collect blood specimens for the same biomarkers for the mother-infant dyad analysis. These assays will be performed at the Nutrition Research Laboratory (NRL) at Aga Khan University. Using the same subsample dyad, we are also planning to perform plasma proteomics to assess the element of antibiotic resistance.

Furthermore, breast milk from the same lactating women will be collected to assess the quality of breast milk composition (macro- and micronutrients), human milk oligosaccharides (HMOs), immunoglobulins, and microbiome analysis. The analysis of breastmilk specimens will be performed in the Azad Lab at the University of Manitoba. A material transfer agreement (MTA) will be developed with the University of Manitoba for the shipment of the specimens.

We will also collect stool specimens at the same time points from the same women and their infants, i.e., 50 pairs per arm. We will assess inflammatory biomarkers in the stool, such as calprotectin (μg/gm), lipokalin-2 (pg/ml), and myeloperoxidase (MPO), using ELISA. Furthermore, the stool samples will also be analyzed to detect enteropathogens using the TaqMan Array Card (TAC) system for polymerase chain reaction (PCR), which will be performed in the Infectious Disease Research Lab (IDRL). Moreover, we will perform targeted Bifidobacterium identification using real-time PCR at IDRL. We will also send the stool samples to the University of Stanford after signing the MTA for additional metagenomic analyses.

The specimens included for further analysis and for future research (indefinite time) will be stored at the IDRL and NRL storage areas at Aga Khan University in − 80 °C freezers. The samples will be deidentified with barcodes, specific IDs for different time points, and mother-infant dyad information and color coded by the type of specimen as part of a unique identification system. All the ethical aspects pertaining to the storage of these samples have been approved by the Ethics Review Committee at Aga Khan University.

### Monitoring and quality assurance

There will be a specific team from Aga Khan University with expertise in data management and trial implementation working with the investigators, and this team will be responsible for general trial processes, such as auditing the trial data and processes to ensure the completeness and accuracy of the protocols, and training the research staff and outcome assessors. Furthermore, there will be independent experts who will make visits for monitoring. A mechanism has also been developed for the research teams to report weekly progress on key progress indicators with the investigators at the VITAL Pakistan Trust and Aga Khan University. Furthermore, the astringent quality assurance mechanism was developed through 10% of the data being checked by trial supervisors and associates. All research teams have received Good Clinical Practice certifications. Comprehensive training and refreshers will be conducted on a routine basis. The anthropometric measurements will be standardized, and the team members will be trained by WHO-trained master trainers.

### Data Safety and Monitoring Board

The independent group of experts, comprising 5 members, constitutes the Data Safety and Monitoring Board (DSMB) for the trial and are responsible for monitoring safety indicators, adverse events, and the results of the interim analysis. The interim analysis (blinded by arm) is scheduled for when 50% of the enrolled participants complete the 6-month follow-up. Only DSMB members will have access to the results of interim analysis, which will be shared by the independent statistician. Data on severe adverse events will be shared with the board on a monthly basis in the form of a progress report.

### Participant safety

Close follow-up will be performed to ensure participant safety, and both the lactating women and the infants will be referred if needed to physicians at the primary health care clinic, with facilitated referral to tertiary hospitals when required. An independent DSMB will monitor the safety of the trial participants and provides trial oversight. Monthly reports on severe adverse events will be shared with the DSMB, and when a safety signal is observed, the DSMB may stop this trial prior to the completion of recruitment. The safety net, including facilitated referral and reporting, is believed to minimize the chances of harm to participants.

### Possible risks

There may be diarrhea, nausea, vomiting, skin rash, or abdominal distension after the use of BEP. Similarly, the infants may develop diarrhea, nausea, vomiting, skin rash, and abdominal distension after the azithromycin dose. We will systematically collect information on all adverse events at each follow-up by asking about the history of any illness since the past visit and assessing signs of concern at each visit. If the mother and infant are considered to have any sign or symptom that is concerning, there is a referral mechanism in place. A 24/7 phone number is provided, and the participant can call this number for any kind of illness; immediate referral will be arranged. This information is well documented and recorded under adverse event reporting. For reporting purposes to the ethics committee and DSMB, we specifically divided “severe adverse events” into two main categories. The first category is “fatal events,” which includes all fatal events, regardless of the underlying cause, occurring among participants (mother and infant) during the follow-up period, and the second category is “nonfatal events,” where study participants (mother and infant) require hospitalization or receive injectable therapy for any illness or diagnosis. Risk management includes prevention through rigorous follow-ups, continuous monitoring of danger signs, documentation, and prompt referrals. Every illness or danger sign reported or identified is addressed through facilitated referral for both women and infants.

### Statistical analysis

Statistical analysis will be performed by Stata, version 15. The baseline characteristics will be assessed by arm. The primary analysis will be an intention-to-treat (ITT) analysis. We will compare the mean length velocity (cm/month) (primary outcome), weight velocity (gm/kg/day), LAZ, WLZ, and WAZ between the two intervention arms and the control arm using repeated measures ANOVA, with the model adjusted for the birth weight and age of the infants at enrollment. If an outcome is missing for the intention-to-treat infants, the means from that group will be imputed. Subgroup analysis will be performed according to the maternal BMI and MUAC categories and compliance with the intervention.

### Participants and public involvement

The investigators have extensive experience working with the community and their representatives/elders. During the protocol development phase, the team discussed and received feedback on the research question and trial design from the community representatives. Furthermore, community perspectives about the trial procedures, especially the frequency and duration of follow-up and biospecimen collection procedure, were also collected. Additionally, during the pilot phase, the aim was not only to test the consent and questionnaires but also to understand how the community responds to different questions and how sensitive information regarding the antenatal and postnatal periods can be collected in a receptive and profound manner.

## Discussion

This trial will provide evidence on the impact of nutrient supplementation in malnourished lactating women in low- and middle-income settings with high breastfeeding rates for the improvement of stunting and growth at 6 months of age in infants. Dissemination and promotion of trial results will occur through small- and medium-scale communication events with stakeholders as well as symposia, publications, and websites that will favor the exchange of ideas.

### Strengths and limitations of this study

The study was uniquely designed from the perspective that robust data on nutritional interventions are grossly lacking for malnourished mothers during periods of exclusive breastfeeding, especially data on the impact of interventions combined with a prophylactic dose of azithromycin to the infant. Furthermore, we foresee some potential biases affecting the results of our trial, which may impose some limitations; they are listed below with countermeasures.

1. Selection bias: Selection bias can occur due to the unblinded nature of the study. This bias has been mitigated by independent allocation sequence generation and block randomization in a sealed opaque envelope.
2. Performance bias and detection bias: The blinding of the outcome assessors to the study arms through independent teams will decrease these biases.
3. Incomplete outcome data (attrition bias): The period of follow-up is 6 months, which is substantially long and leads to a risk of participants being lost to follow-up. However, efforts will be made to mitigate this through communication with the community in the trial run-in period and connecting with families. If a participant moves away from the catchment area, if possible, they will be followed at their new address until the outcome of the trial. Regardless, we will use all available data during analysis, even if there is missing data at certain time points.
4. Noncompliance bias: Noncompliance can be caused by the taste of the supplements and long duration of consumption, which can lead to noncompliance with the intervention. This will be addressed through detailed counseling at the time of consent and providing potential study participants 1 to 2 days for decision-making and/or seeking the input of the household decision-makers. Similarly, in Pakistan, where the prevalence of exclusive breastfeeding is between 35 and 40%, the continuous assurance by the team that infants should be on exclusive breastfeeding for 6 months is also very challenging. To address this concern, continuous counseling throughout the arm is the key, which will be performed at the follow-ups.
5. Contamination: Contamination will be prevented by enrolling only one participant from a single household and through the provision of excess supplements, as women are likely to feed the supplements to other members of the family.

## Conclusion

The findings of the trial will help in reshaping the policies pertaining to the prevention of undernutrition among lactating women and the resulting poor growth of infants under 6 months of age. There will be significant policy implications for the global problem of undernutrition among infants for constrained health systems with a high burden of attributable infant deaths.

## Trial status

Active protocol version number: 1.3; January 16, 2019. Protocol amendments have been submitted, and the details of the protocol versions with the date of the amendment are provided in Table 3. Recruitment began on August 1, 2018. Currently, recruitment is ongoing and is expected to be completed in June 2020, and the last follow-up is expected to be completed in November 2020. The database will be locked in January 2021.

**Table 3:** Protocol versions

Version	Date and changes
1.0	January 16, 2018—original protocol.
1.1	July 22, 2018—introduction and background was improved.
1.2	September 5, 2018—amendments of the inclusion and exclusion criteria.
1.3	January 16, 2019—more comprehensive plan of the analysis was incorporated.

## Data Availability

Processes will be developed to facilitate data sharing for scientific purposes in a collaborative manner. Deidentified data with analytical/statistical codes will be available in the public domain 2 years after the publication of the main manuscripts with investigator support after the approval of the proposal and the data access agreement has been signed. It will be available to researchers whose proposed use of the data has been approved. The data will be uploaded on a password-secure cloud server.

## Abbreviations

AGP: Alpha1-acid glycoprotein
BEP: Balanced energy protein
BMI: Body mass index
CRP: C-reactive protein
CRF: Case report form
DSMB: Data Safety and Monitoring Board
ERC: Ethics Review Committee
HMO: Human milk oligosaccharides
IFA: Iron-folic acid
IRB: Institutional Review Board
ITT: Intention-to-treat
IDRL: Infectious Disease Research Lab
LAZ: Length-for-age *z*-score
LW: Lactating women
LMICs: Low- and middle-income countries
MTA: Material Transfer Agreement
MPO: Myeloperoxidase
MMN: Multiple micronutrient
MUAC: Mid-upper arm circumference
NRL: Nutrition Research Lab
PCR: Polymerase chain reaction
SQ-LNS: Small quantity lipid-based nutrient supplements
TAC: TaqMan Array Card
WAZ: Weight-for-age *z*-score
WLZ: Weight-for-length *z*-score
